# Influence of the constraint-induced method of constraint-induced movement therapy on improving lower limb outcomes after stroke: A meta-analysis review

**DOI:** 10.3389/fneur.2023.1090808

**Published:** 2023-03-16

**Authors:** Jing Zhang, Hongsheng Feng, Jinpeng Lin, Hua Zhai, Xia Shen

**Affiliations:** ^1^Key Laboratory of Exercise and Health Sciences of Ministry of Education, Shanghai University of Sport, Shanghai, China; ^2^Shanghai Yangzhi Rehabilitation Hospital (Shanghai Sunshine Rehabilitation Center), School of Medicine, Tongji University, Shanghai, China; ^3^School of Materials Science and Engineering, South China University of Technology, Guangzhou, China; ^4^Department of Administration, Shanghai Yangzhi Rehabilitation Hospital (Shanghai Sunshine Rehabilitation Center), Tongji University School of Medicine, Shanghai, China; ^5^Rehabilitation Medicine Research Center, Shanghai Yangzhi Rehabilitation Hospital (Shanghai Sunshine Rehabilitation Center), School of Medicine, Tongji University School of Medicine, Shanghai, China

**Keywords:** stroke, paresis, lower limb, constraint-induced movement therapy, ICF, meta-analysis

## Abstract

**Background:**

Constraint-induced movement therapy (CIMT) targeting the lower limb function uses various methods. The influence of CIMT methods on lower limb outcomes after stroke has rarely been examined.

**Objectives:**

This study aimed to examine CIMT effects on lower limb outcomes and explore the influence of CIMT methods on treatment effects after stroke, with other potential factors considered as covariates.

**Methods:**

PubMed, Web of Science, Cochrane Library, Academic Search Premier *via* EBSCOHost, and PEDro databases were searched until September 2022. We included randomized control trials with CIMT targeting the lower limb function and dosage-matched active control. The Cochrane risk-of-bias tool was used to evaluate the methodological quality of each study. Hedges' g was used to quantify the effect size of CIMT on outcomes compared to the active control. Meta-analyses were conducted across all studies. A mixed-variable meta-regression analysis was used to investigate the influence of CIMT methods on treatment effects after stroke, with other potential factors considered as covariates.

**Results:**

Twelve eligible randomized controlled trials with CIMT were included in the meta-analysis, where 10 trials were with a low risk of bias. A total of 341 participants with stroke were involved. For the treatment effects on the lower limb function, CIMT showed a moderate short-term effect size [Hedges' g = 0.567; *P* > 0.05; 95% confidence interval (CI): 0.203–0.931], but a small and insignificant long-term effect size (Hedges' g = 0.470; *P* > 0.05; 95%CI: −0.173 to 1.112), compared with conventional treatment. The CIMT method of using a weight strapped around the non-paretic leg and the ICF outcome category of the movement function were identified as significant factors contributing to the heterogeneity of short-term effect sizes across studies (β = −0.854 and 1.064, respectively, *R*^2^ = 98%, *P* > 0.05). Additionally, using a weight strapped around the non-paretic leg had a significant contribution to the heterogeneity of long-term effect sizes across studies as well (β = −1.000, *R*^2^ = 77%, *P* > 0.05).

**Conclusion:**

Constraint-induced movement therapy is superior to conventional treatment for improvement of lower limb function in the short-term but not in the long-term. The CIMT method of using a weight strapped around a non-paretic leg contributed negatively to the treatment effect, and therefore might not be recommended.

**Systematic review registration:**

https://www.crd.york.ac.uk/PROSPERO, identifier: CRD42021268681.

## 1. Introduction

Stroke, a common public health problem, is a leading cause of death and disability in adults (1). The mortality rate decline, especially in high-income countries, has been attributed to the continuous implementation of evidence-based stroke prevention strategies (2); however, most stroke survivors suffer from long-term impairment, activity limitation, and participation restriction (1). Evidence-based stroke rehabilitation treatment ensures the effectiveness of optimizing function, reducing disability, enabling social participation, and improving the quality of life for survivors (3).

Constraint-induced movement therapy (CIMT) is a treatment regimen that facilities the use of the upper paretic limb by constraining the non-paretic upper extremity, using mass task practice with the paretic limb, and transfer package (4). CIMT improves the outcome of the upper paretic limb and is categorized as class IIa recommended evidence-based treatment in stroke rehabilitation guidelines (3, 4). Therefore, CIMT with a constraint on the non-paretic lower limb has been designed to facilitate the use of the lower paretic limb. Unlike the upper limb, the constraining methods for the non-paretic lower limb are not standardized. Completely constraining the non-paretic lower limb is impossible because functional activities of the lower limbs are bipedal. Therefore, various lower limb CIMT methods, such as using a long-leg orthosis or weight strapped around the non-paretic leg to constrain its movement (5–8), using a wedged insole under the non-paretic foot to constrain over-weight-bearing of the non-paretic leg (5, 9) or task-specific force-use therapy for paretic leg were developed (10–13). Moreover, the upper limb constraint method was also used to improve the lower limb function, which produces therapeutic effects by restricting trunk movement and interfering with equilibrium (14). It is uncertain which is superior for improving lower limb outcomes.

With increasing evidence that CIMT improves lower limb outcomes in stroke survivors, meta-analysis is the optimal method to explore the influence of CIMT methods on the therapeutic effects of lower limbs. Two relevant meta-analysis reviews have been published (15, 16). These studies divided articles into different subgroups based on outcome measures; Tedla et al. (15) further split articles based on outcome measures and the CIMT method. The subgroup method reduced the number of studies and the power of statistical tests in the meta-analysis, leading to unexplained large heterogeneity (17). Moreover, the CIMT methods in the review were only categorized into upper and lower limb constraints. The effect size had heterogeneity across studies with constraints at the lower limb, but it was unclear how the constraint methods influenced the effect size. Furthermore, Tedla et al. focused on two contributing factors of treatment effects: types of outcome measures and CIMT methods; however, they did not perform statistical analysis between factor, and were therefore unable to avoid occasional results contributed by other factors.

Therefore, this study aimed to examine the effects of CIMT on lower limb outcomes and explore the influence of CIMT methods on treatment effects after stroke, with other potential factors considered as covariates.

## 2. Methods

### 2.1. Search strategy

This review was conducted following the “Preferred Reporting Project for Systematic Evaluation and Meta-Analysis” (PRISMA) guidelines (18). Five databases were selected for the literature search, including PubMed, Web of Science, Cochrane Library, Academic Search Premier *via* EBSCOHost, and PEDro. The keywords used to conduct the literature search were combined with the following English terms: “stroke OR apoplexy OR cerebrovascular accident” AND “constraint-induced movement therapy OR modified constraint-induced movement therapy OR CIMT OR mCIMT OR force use” AND “lower limbs OR lower limb OR lower extremity OR lower extremities”. The language was restricted to English. We further checked the reference lists of identified articles to discover other potential studies. The literature search was performed up to 30 September 2022.

### 2.2. Eligibility criteria and the selection process

The inclusion criteria included are as follows:

1) Studies having patients with stroke, with age ≥ 18 years selected as participants;
2) Randomized controlled trials that involved experimental group(s) receiving CIMT targeted at the motor function, balance, and mobility of the lower limb, and an active control group with dose-matched conventional intervention without CIMT or with different CIMT interventions.

The exclusion criteria involved are as follows:

1) With pure control groups without matched intervention because the effect on outcomes is attributed to the additional intervention and not the CIMT method (19);
2) Without a clear description of the study design, measurement procedure, and intervention protocol, or with unreported relevant results.

Two researchers (the first and second authors) determined the eligibility of studies by screening the title, abstract, and full text. Any divergence related to trial eligibility was resolved by discussion with a third party (the third author).

### 2.3. Data collection process and data items

Two researchers developed a data collection sheet together, then worked on the data collection independently, and afterward checked accuracy together. Extracted information from each study included the characteristics of the participants, intervention and outcome measures, and the data of outcome measures at each assessment interval. The participant characteristics extracted included sample size, age, sex, time after the stroke onset, sides of the brain lesion and stroke type, and cognitive condition if available. The intervention dosage extracted included total training hours and training weeks. Participant characteristics in each study were calculated through addition or estimated by the mean value of each group corrected from the sample size in each group because most studies separately reported these characteristics in the experimental and control groups. Furthermore, the CIMT method, the matching situation of training tasks between CIMT and conventional interventions, and training dosage in total time (hours) and duration (weeks) were extracted. In some studies where the dosage was quantified by the number of practice repetitions, 300 repetitions were converted to 1 h (20).

For the outcome measures, based on the framework of the International Classification of Functioning, Disability, and Health (ICF), they were categorized as movement function, activity performance or independence, activity participation, or quality of life (21). For assessment with a follow-up period after the intervention, the duration of follow-up was extracted. The mean and standard deviation of each outcome measure at each assessment interval of the experimental and control groups were collected as well.

### 2.4. Study risk-of-bias assessment

The methodological quality of each study was evaluated by two researchers independently using the Cochrane risk-of-bias tool (22). The Cochrane risk-of-bias tool comprises seven items, including random sequence generation, allocation concealment, blinding of participants and personnel, blinding of outcome assessment, incomplete outcome data, selection, and other sources of bias. Each item includes three outcomes: low, high, and unclear risks of bias. Disagreement on the scoring between the two researchers was resolved *via* discussion with the corresponding author.

### 2.5. Effect measures and synthesis methods

Considering that the sample sizes of included studies were relatively small, Hedges' g which corrects for small sample sizes (23) was used to quantify the effect size of CIMT compared with the control group. It was calculated by dividing the raw difference in mean change between the CIMT experiment and control groups by the estimated pooled standard deviation of the changes and then adjusting for bias due to sample size in each group. In one included study with two CIMT groups and one active control group, the active control group was included two times in meta-analysis comparisons. For each comparison, the sample size of the active control group was divided equally (15). Positive values of Hedges' g indicate effects in favor of CIMT or otherwise in favor of the active control. The effect sizes were set at 0.2, 0.5, and 0.8, corresponding to small, moderate, and large effects.

Based on the two kinds of active controls (conventional treatment or CIMT) and the two kinds of post-treatment assessment intervals [short-term (immediately after treatment) or long-term (follow-up after treatment)], there were four kinds of study designs in the meta-analyses. Meta-analyses were conducted across all studies of the same design first. Only one outcome measure of each study was included in each meta-analysis, which appeared most frequently in included studies of the corresponding meta-analysis (24, 25). Considering ICF categories of outcome measures showing significant contributions to the effect sizes found in the present study (reported in the section Results), meta-analyses across studies with the same design but grouped based on ICF categories of outcome measures were further conducted. The second kind of meta-analysis insured the maximum number of studies included when exploring the CIMT effects on outcomes of each ICF category, the number of studies were equal to or more than the number in the first kind of meta-analyses, which enabled a clearer view when further investigating the influence of CIMT methods on treatment effects.

I^2^ statistics were used to assess statistical heterogeneity across studies in each meta-analysis, and an I^2^ of 25, 50, or 75% was considered as low, moderate, or high heterogeneity, respectively. If there was insignificant heterogeneity across studies, the fixed-effects model was used to analyze the training effects; otherwise, the random-effects model of the meta-analysis was adopted.

For significant heterogeneity across studies of each meta-analysis, publication bias was first evaluated using Egger's test to determine any association between the effect size and sample size. Subsequently, meta-regression or subgroup analysis was used to analyze the contributing roles of the potential factors to the heterogeneity. CIMT features in experimental groups were included as the potential factors of interest in the regression analysis as well as participant characteristics and ICF categories of outcome measures. The factor of ICF categories was only included in the meta-regression across all studies. The meta-regression analysis has three steps with reference to the procedure of multiple linear regression (26). First, identifying potential factors with significant contributions in the single-variable model. Second, significant factors identified at the first step were entered into the first mixed-variable regression model to further identify factors with a significant contribution in the mixed-variable regression model or with a significant change by the test of change. Finally, identified factors in the second step were entered into the final mixed-variable regression model. The association between each potential significant factor was analyzed using Spearman rank correlation (significant Statistical Product and Service Solutions version 25.0) before entering them into the first mixed-variable regression model. If two factors are strongly correlated with each other (*r* > 0.7), only the factor with the higher proportion of variance in the single-variable model was entered into the first mixed-variable regression model. A subgroup analysis was used to investigate factors contributing to heterogeneity when the number of studies was insufficient to conduct a meta-regression (27).

Comprehensive meta-analysis software (Version 3, Biostat, Englewood, New Jersey) was adopted to conduct meta-analyses. Statistical significance was set at *P* < 0.05 for all tests except for the single-variable regression model with *P* > 0.10 to identify potential factors for the mixed-variable regression model.

## 3. Results

### 3.1. Study selection, study characteristics, and methodological quality

**Figure 1:**
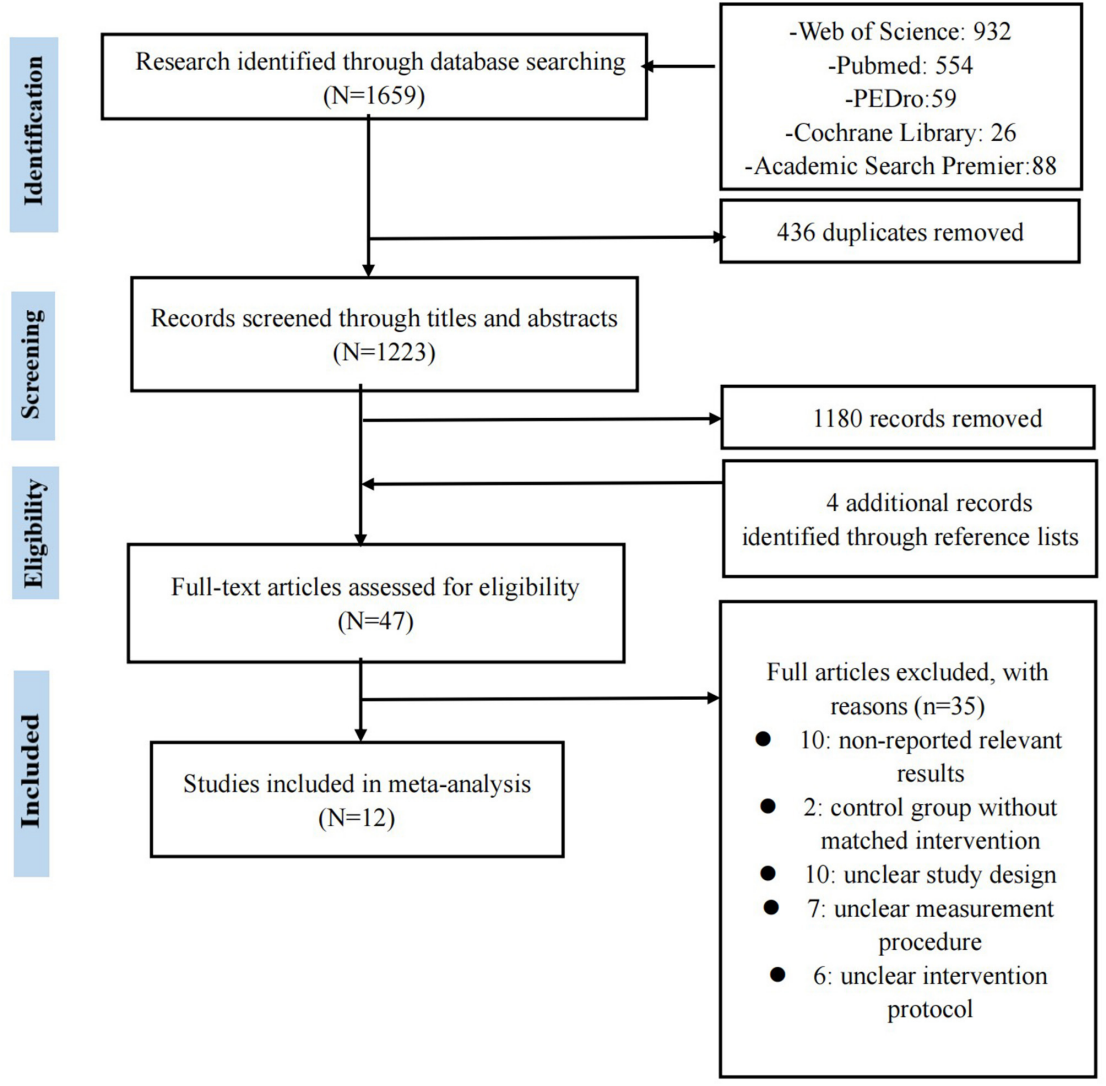
PRISMA flow diagram showing flow of information through the review.

We identified 1,659 studies *via* database searches, and four additional studies were identified by checking reference lists in identified articles. Figure 1 presents the details of the trial flow. The qualitative synthesis included 12 studies involving 341 participants.

For participants in included studies, the mean age ranged from 49 to 62, the ratio of male to female ranged from 0.8 to 3.5; the mean time after the onset ranged from 1.9 to 80.4 months; the affected side ratio of right to left ranged from 0.4 to 2.5; and the percentage of ischemic stroke ranged from 80 to 100%. In studies that reported the score of mini-mental state examination (7, 10, 11) or set no cognitive impairment as the selection criterion, participants had no cognition impairment (6, 12–14, 28, 29).

Five CIMT methods were ranked by frequency of using in the included studies, as below:

1) Task-specific force-use therapy focusing on the paretic leg (10–13),
2) Using an orthosis or brace to constrain non-paretic leg movement (5, 6, 8),
3) Using an arm sling to constrain non-paretic arm movement (14, 28, 29),
4) Using a wedged insole under the non-paretic foot to constrain over-weight-bearing of the non-paretic leg (5, 9),
5) Using a weight strapped around the non-paretic leg to constrain non-paretic leg movement (7).

For the CIMT dosage in included studies, the total training time ranged from 5 to 60 h, excluding CIMT time in daily life, which ranged from 42 to 460 h (14). The training duration ranged from 1 day to 5 weeks. Three studies facilitated the transfer of learned skills from CIMT into daily activities but did not report the transfer dosage (5, 6, 11).

For outcome measures, the walking test had the highest frequency in all measures and also in measures of the activity performance category, followed by the Berg Balance Scale (BBS). The Fugl-Meyer Assessment of Lower Extremity (FMA-LE) had the highest frequency in measures of the movement function category. In a single study, the stroke impact scale was uniquely related to participation or the quality of life (14). Four studies conducted a patient assessment at follow-up after treatment. The follow-up period ranged from 4 to 12 weeks (7, 9, 11, 12, 14). Table 1 presents the detailed characteristics of each study.

**Table 1:** Characteristics of all included studies.

**Studies**	**Subjects**				**Intervention**				**Assessment**	
**References**	**Sample sizes**	**Sex (male/female); Age (mean)**	**Type (Hemor /Isch); Paretic side (R/L); Time after stroke onset (mth)**	**Cognition status**	**CIMT manner in EXP group**	**CIMT training protocol in EXP group**	**Training protocol in CON group**	**Treatment total h; total wks**	**Outcome measures**	**Follow-up duration (Week)**
Aloraini (11)	CIMT:19 CON:19	CIMT: 10/9; 60.1 ± 10.8 yrs CON: 9/10; 59.3 ± 11.4 yrs	CIMT: 3/16; 6/13; 30.2 ± 13.9 mth CON: 4/15; 8/11; 36.8 ± 19.5 mth	MMSE: CIMT:27.5 ± 1.7 CON: 27.3 ± 1.7	Task-specific force-use therapy focusing on paretic leg	Functionally-oriented physical exercises toward the more effected lower extremity; Encouraging transfer in daily life	Unmatched training protocol as the EXP group; including range of motion and stretching exercises, balance, walking and endurance training	35 h; 2wks	10MWT,# BBS,# FMA-LE,@ 6MWT.#	12
Abdullahi et al. (16)	CIMT:30 CON:28	CIMT:12/18; 50.2 ± 13.9 yrs CON: 13/15; 47.8 ± 14.7 yrs	CIMT: 5/25; 20/10; 8 ± 14.9 mth CON: 3/25; 16/12; 8.5 ± 13 mth	MMSE: CIMT:27 ± 2 CON: 28 ± 3	Task-specific force-use therapy focusing on paretic leg	Training tasks including stepping forward, backward stepping, side stepping, ball kicking, and stair climbing; With dosage quantified by repetitions; 2/5 training performed at home	CIMT training as well with same task with different dosage quantified method by time duration; 2/5 training performed at home	12,000 repetitions/60 h; 4 wks	10MWT,# BBS,# FMA-LE,@ 6MWT,# RMI,# Perceived Exertion@	0
Acaroz et al. (5)	CIMT:15 CON:15	CIMT: 8/7; 55.13 ± 14.7 yrs CON: 6/9; 57.67 ± 12.2 yrs	CIMT: 4/11; 5/10; 6.8 ± 2.7 mth CON: 3/12; 5/10; 6.6 ± 3.2 mth	Nil	Using wedged insole under non-paretic foot to constrain over-weightbearing of non-paretic leg; Using a long-leg orthosis to constrain the movement of non-paretic leg	A series of functional activities including sit-to-stand, weight bearing, climbing stairs and ramp, balance activities, stepping over obstacles, treadmill walking etc. Encouraging transfer in daily life	Unmatched protocol with the EXP group, that is Neurodevelopmental therapy including ROM, strengthening, weight-bearing, balance activities, and walking etc.	20 h; 2 wks	10MWT,# BBS,# FAC,# Step lengthasymmetry,# Postural symmetry ratio.#	0
Choi et al. (12)	CIMT1:12 CIMT2:12 CON:12	CIMT1: 7/5 61.25 ± 5.59 yrs CIMT2: 6/6; 62.58 ± 5.51 yrs CON: 8/4; 61.92 ± 6.08 yrs	CIMT1: nil; 7/5; 13.8 ± 3.9 mth CIMT2: nil; 3/9; 13.6 ± 5.5 mth CON: nil; 4/8; 14.3 ± 4.8 mth	No cognitive impairment that based on MMSE scores as one selection criterion.	Task-specific force-use therapy focusing on paretic leg	Game exercises including Ski slalom and Soccer heading game	Matched game exercises	6 h; 4 wks	TUG,# FRT,# mFRT,# COP sway amplitude/ velocity/ area.@	0
Danlami and Abdullahi (6)	sCIMT:5 tCIMT:6 CON:7	sCIMT: 10/9; 48.2 ± 7.89 yrs tCIMT: 2/4; 55.67 ± 9 yrs CON: 6/1; 54.14 ± 6.87 yrs	sCIMT: 1/4; 2/3; 1.4 ± 1.1 mth tCIMT: 2/4; 2/4; 2.5 ± 1.9 mth CON: 2/4; 5/2; 2.5 ± 1.6 mth	No cognitive impairment (MMSE≥17) as one selection criterion.	Using a knee orthosis to constrain the movement of non-paretic leg;	Same tasks in two CIMT training groups, including sit-to-stand, forward and backward stepping, stair climbing and descending, side-to-side stepping; Different dosage quantified methods, one by repetition, the other by time duration; Encourage use in daily life (90% waking hour)	Unmatched training protocol with the EXP groups, including passive movement, therapeutic positioning, strengthening exercise and over-ground gait training.	9,600 repetitions/40 h; 4 wks	FMA-LE.@	0
eSilva et al. (7)	CIMT:19 CON:19	CIMT: 13/6; 57.37 ± 9.22 yrs CON: 10/9; 57.44 ± 15.93 yrs	CIMT: 5/14; nil; 3 ± 4.4 mth CON: 1/18; nil; 3 ± 3.7 mth	MMSE: CIMT:24 ± 3.7 CON: 23 ± 3.0	Using weight strapped around non-paretic leg to constrain the movement of non-paretic leg	Treadmill training	Matched treadmill training	9 h; 2 wks	BBS#, TUG#, Turn performance.#	6
Silva-Filho and Albuquerque (28)	CIMT:9 CON:10	CIMT: 6/3; 52 ± 5.5yrs CON: 5/5; 59.5 ± 4.3yrs	CIMT: 3/6; 8/1; 13.7 ± 8.4mth CON: 1/9; 6/4; 29.3 ± 24.7 mth	No cognitive impairment that based on MMSE scores as one selection criterion.	Using an arm sling to constrain non-paretic arm	Only paretic upper limb specific training, but no lower limb specific training. Use in daily life	Matched training protocol with the experimental group	72 h in daily life; 4 wks	Gait velocity,# BBS,# TUG,# Stairs up and downs. #	0
Jung et al. (13)	CIMT:11 CON:10	CIMT: 7/4; 56.4 ± 11.1 yrs CON: 7/3; 56.3 ± 17.1 yrs	CIMT: 3/8; 3/8; 6.2 ± 2.5 mth CON:2/8;3/7; 7 ± 2.5 mth	No cognitive impairment (MMSE≥24 scores) as one selection criterion.	Task-specific force-use therapy focusing on paretic leg	Gait training under augmented cues	Matched training protocol with the experimental group	10 h; 4 wks	Gait velocity,# Force of the cane,# Support of affected side,# Muscle activation.@	0
Kim and Cha (29)	CIMT:10 CON:10	CIMT: 6/4; 57.6 ± 3.7 yrs CON: 7/3; 51.9 ± 6.1 yrs	CIMT: nil; 3/7; 24.1 ± 10.7 mth CON: nil; 6/4; 30.8 ± 11.0 mth	No cognitive impairment (MMSE≥24 scores) as one selection criterion.	Using an arm sling to constrain non-paretic arm	Ground gait training	Matched Ground gait training as the EXP group	6 h; 4 wks	TIS-dynamic,@ TIS-coordination,@ TIS-static,@ LOS-affected side,@ LOS-unaffectedside.@	0
Aruin et al. (9)	CIMT:9 CON:9	Overall: 14/4; 57.7 ± 11.9 yrs	Overall: nil; 9/9; 80.4 ± 46.8 mth	Nil	Using wedged insole under non-paretic foot to constrain over-weightbearing of non-paretic leg	Weight bearing, sit-to-stand, balance exercises, and walking exercise; Use in daily life	Matched training protocol with the experimental group	6 h training, +42 h in daily life; 6 wks	Gait velocity,# BBS,# FMA-LE,@ Weight bearing.@	12
Fuzaro et al. (14)	CIMT:19 CON:18	CIMT: 12/7; 54.15 ± 12.94 yrs CON: 9/9; 50.78 ± 15.65 yrs	CIMT: nil; 14/5; 19.6 ± 20.9 mth CON: nil; 9/9; 30.8 ± 31.8 mth	No cognitive impairment that based on MMSE scores as one selection criterion.	Using an arm sling to constrain non-paretic arm	No any training task except for upper limb mobilization; Use in daily life	The other CIMT training but with task-specific force-use therapy focusing on paretic arm. Thus, it was regarded as control with conventional care on lower limb.	460 h in daily life; 4 wks	10MWT,# BBS,# TUG,# FMA,@ Stroke ImpactScale. ^∧^	12
Gatti et al. (8)	CIMT:5 CON:5	Overall: 6/4; 55.5 ± 12.9 yrs	Overall: 0/10; 4/6; 1.9 ± 0.9 mth	Nil	Using a knee orthosis to constrain the movement of non-paretic leg	Training with shaping activities;	Matched training protocol with the experimental group	6 h; 0.1 wk (1 day)	Gait velocity (stride speed),# Stride length#, Swing phase asymmetry index#.	0

EXP, experimental group; CON, control; CIMT, constraint-induced movement therapy; Hemor /Isch, hemorrhagic /ischemic; R/L, right/left; MMSE, mini mental state examination; 10MWT, 10-meter walking test; 6MWT, 6-minute walking test; BBS, Berg balance scale; FAC, functional ambulation classification; FMA-LE, Fugl-Meyer Assessment of the lower limb; LOS, limit of stability; LOS, limit of stability; TIS, Trunk Impairment Scale; TUG, timed up and go test; yrs, years; mth, months; h, hours; wks, weeks.

#, outcome measures related to activity performance/independence; @, outcome measures related to the movement function (motor function and posture); ^∧^, outcomes related to participation/ the quality of life.

For methodological quality, most studies showed moderate methodology quality with a low risk of bias in three or more items of the Cochrane risk-of-bias tool, except for two studies (Table 2) (9, 14).

**Table 2:** Cochrane risk-of-bias of articles included in the meta-analysis.

**References**	**Random sequence generation**	**Allocation concealment**	**Blinding of participants and personnel**	**Blinding of outcome assessment**	**Incomplete outcome data**	**Selective reporting**	**Other sources of bias**	**Quality^∧^**
Aloraini (11)	Low	Low	High	Low	Low	Unclear	Unclear	Good
Abdullahi et al. (16)	Low	Low	Low	Low	Low	Unclear	Unclear	Good
Choi et al. (12)	Low	Low	High	Low	Low	Unclear	Unclear	Good
eSilva et al. (7)	Low	Low	High	Low	Low	Unclear	Unclear	Good
Danlami and Abdullahi (6)	Low	Low	High	Low	Low	Unclear	Unclear	Good
Acaroz and Livanelioglu (5)	Low	High	High	Low	Low	Unclear	Unclear	Good
Kim and Cha (29)	Low	Unclear	High	Unclear	Low	Unclear	Unclear	Poor
Fuzaro et al. (14)	Low	Low	High	Low	Unclear	Unclear	Unclear	Good
Aruin et al. (9)	Low	Unclear	High	Unclear	Low	Unclear	Unclear	Poor
Jung et al. (13)	Low	Low	High	Low	Low	Unclear	Unclear	Good
Silva-Filho and Albuquerque (28)	Low	Low	High	Low	High	High	Unclear	Good
Gatti et al. (8)	Low	Unclear	High	Low	Low	Unclear	Unclear	Good

^∧^Quality is scaled as good for studies with at least three items at low risk or otherwise as poor.

### 3.2. Short-term effects of CIMT compared with conventional treatment on lower limb outcomes

**Figure 2:**
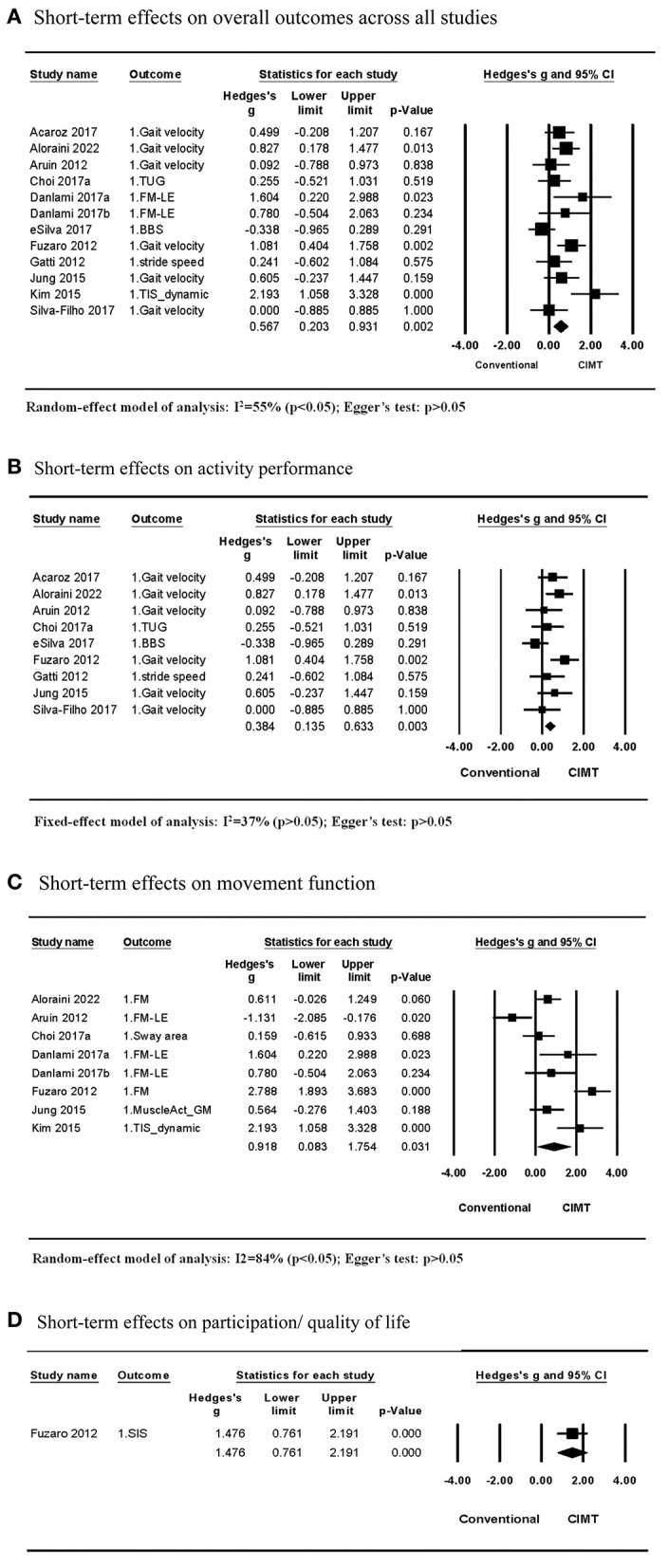
Short-term effects of CIMT compared with conventional treatment. **(A)** Short-term effects on overall outcomes across all studies. **(B)** Short-term effects on activity performance. **(C)** Short-term effects on movement function. **(D)** Short-term effects on participation/quality of life.

Eleven studies (12 comparisons) compared the short-term effects of CIMT and conventional treatment (5–9, 11–14, 28, 29). A meta-analysis of the effect size on the outcome between CIMT and conventional treatment revealed moderate heterogeneity across studies (*I*^2^ = 55%; *P* > 0.05). This result demonstrated a statistical significance in favor of the CIMT group, with a moderate effect size [Hedges' g = 0.567; *P* > 0.05; 95% confidence interval (CI): 0.203–0.931]; (Figure 2A). The final regression model included the CIMT method using a weight strapped around the non-paretic leg and the ICF outcome category as significant contributing factors to explain 98% of the heterogeneity across studies. The indication formula is that effect size = 0.517−0.854^*^ if using weight strapped around the non-paretic leg as the CIMT method + 1.064^*^ if outcome belongs to movement function (Table 3).

**Table 3:** Contributing factors to the heterogeneity of short-term effect sizes on the overall outcome of CIMT compared to conventional treatment using three-step meta-regression.

	**Single variable model**			**1st mixed-variables model**						**Final mixed-variables model**					
	β	***P*** **-value**	*R* ^2^	**If included (Y/N)**	β	***P-*** **value**	*R* ^2^	**Change test**		**If included (Y/N)**	β	***P*** **-value**	*R* ^2^	**Change test**	
								***Q*** **-value**	***P*** **-value**					***Q*** **-value**	***P*** **-value**
**Intercept**	-	-	-		3.107	0.000					0.517	0.000			
**Subjects**							0.96						0.98		
Age(mean)	−0.110	0.097!	0.15	Y	−0.046	0.363		2.75	0.097	N					
Male/female	−0.107	0.716	0.00	N											
time after onset(mean)	−0.000	0.961	0.00	N											
percentage of ischemic stroke	−0.170	0.957	0.00	N											
Paretic side ratio: left/right	0.004	0.991	0.00	N											
**Intervention**															
***CIMT Manner***															
If task-specific force-use therapy	0.000	0.999	0.00	N											
If using a knee/leg orthosis	0.224	0.648	0.00	N											
If using an arm sling	−0.016	0.972	0.00	N											
If using wedged insole	−0.521	0.453	0.00	N											
If using weight strapped	−1.002	0.047^*^	0.44	Y	−0.836	0.021^*^		3.86	0.049*	Y	−0.854	0.017^*^		3.96	0.047^*^
***Conventional treatment protocol***															
If matched with CIMT treatment	0.386	0.343	0.00	N											
***Dosage***															
Total training time	0.004	0.495	0.00	N											
Training duration (week)	0.010	0.597	0.00	N											
**Outcome**															
***ICF category***															
If movement function	1.192	0.007^*^	0.59	Y	0.925	0.031^*^		4.66	0.031^*^	Y	1.064	0.007		7.16	0.007^*^
If activity performance/independ	1.192	0.007^*^	0.59	N^∧^											
If participation/quality of life	-	-	-												
Follow-up duration	-	-	-												

^∧^The potential significant factor was not included into the first mixed-variable model since it is actually the same as another factor that was included in the first mixed-variable model (r = 1).

^*^P < 0.05.

^!^P < 0.10.

Among the 11 studies, nine studies examined effects on the activity outcome, while seven studies (eight comparisons) examined effects on the movement function. The meta-analysis of studies examining effects on the activity outcome revealed that CIMT had a small effect size compared to conventional treatment (Hedges' g = 0.384; *P* > 0.05; 95% CI: 0.135 to 0.633) and low heterogeneity across studies (*I*^2^ = 37%; *P* > 0.05) (Figure 2B). For the small heterogeneity, the CIMT method using a weight strapped around the non-paretic leg is the only significant contributing factor discovered in the single-variable meta-regression analysis, explaining 100% of the variance in effect size across studies (β = −0.857, *R*^2^ = 100%, *P* > 0.05) (Tables 3, 4).

**Table 4:** Contributing factors to the heterogeneity of effect sizes of CIMT compared to conventional treatment in all meta-analyses.

	**Short-term effects**			**Long-term**	
	**Overall**	**Activity outcomes**	**Movement function**	**Overall (Activity outcomes)** ^∧^	**Movement function** ^#^
**Heterogeneity (*I*^2^)**	55%	37%	84%	70%	93%
**Regression model (potential contributing factors: coefficient s of Intercept and identified contributing factors)** ^*^					
**Intercept**	0.517	0.519	0.581	0.757	-
**Subjects**					
Age (mean)					
Male/female					
time after onset (mean)					
percentage of ischemic stroke					
Paretic side ratio: left/right					
**Intervention**					
***CIMT Manner***					
If task-specific force-use therapy					
If using a knee/leg orthosis					
If using an arm sling			1.612		Positive
If using wedged insole					
If using weight strapped	−0.854	−0.857	−1.712	−1.000	Negative
***Conventional treatment protocol***					
If matched with CIMT treatment					
***Dosage***					
Total training time					
Training duration(week)					
***Outcome***					
***ICF category***					
If movement function	1.064				
If activity performance/independ					
If participation/quality of life					
**Follow-up duration**					
Proportion of variance explained by the model (*R^2^*)	0.98	1.00	1.00	0.77	

^*^All models were finalized through three-step meta-regression, as shown in Table 3, except for those marked by ^∧^ and #.

^∧^Single-variable regression analysis since there are not enough studies for the mixed-variable regression analysis.

^#^Analyzed by the between-subgroup analysis since there are not enough studies for the regression analysis.

The meta-analysis of studies examining the effects of CIMT on the movement function detected a large effect size compared with conventional treatment (Hedges' g = 0.918 *P* > 0.05; 95% CI: 0.083 to 1.754) and large heterogeneity across studies (*I*^2^ = 84%; *P* > 0.05) (Figure 2C). The CIMT methods using a weight strapped around the non-paretic leg and an arm sling to constrain non-paretic arm movement were contributing factors in the final regression model, explaining 100% of heterogeneity of effect size across studies (*R*^2^ = 100%). The model indicated that effect size = 0.581 – 1.712 ^*^ if using a weight strapped around the non-paretic leg as the CIMT method + 1.612 ^*^ if using an arm sling as the CIMT method (Table 4).

Only one study by Fuzaro et al. examined the effects on participation or the quality of life. They discovered that CIMT had a larger effect size than conventional treatment (Hedges' g = 1.476; *P* > 0.05; 95% CI: 0.761 to 2.191) (Figure 2D).

Egger's test revealed no publication bias across studies included in each meta-analysis on short-term effects (*P* > 0.05) (Figure 2).

### 3.3. Long-term effects of CIMT compared with conventional treatment on outcomes

**Figure 3:**
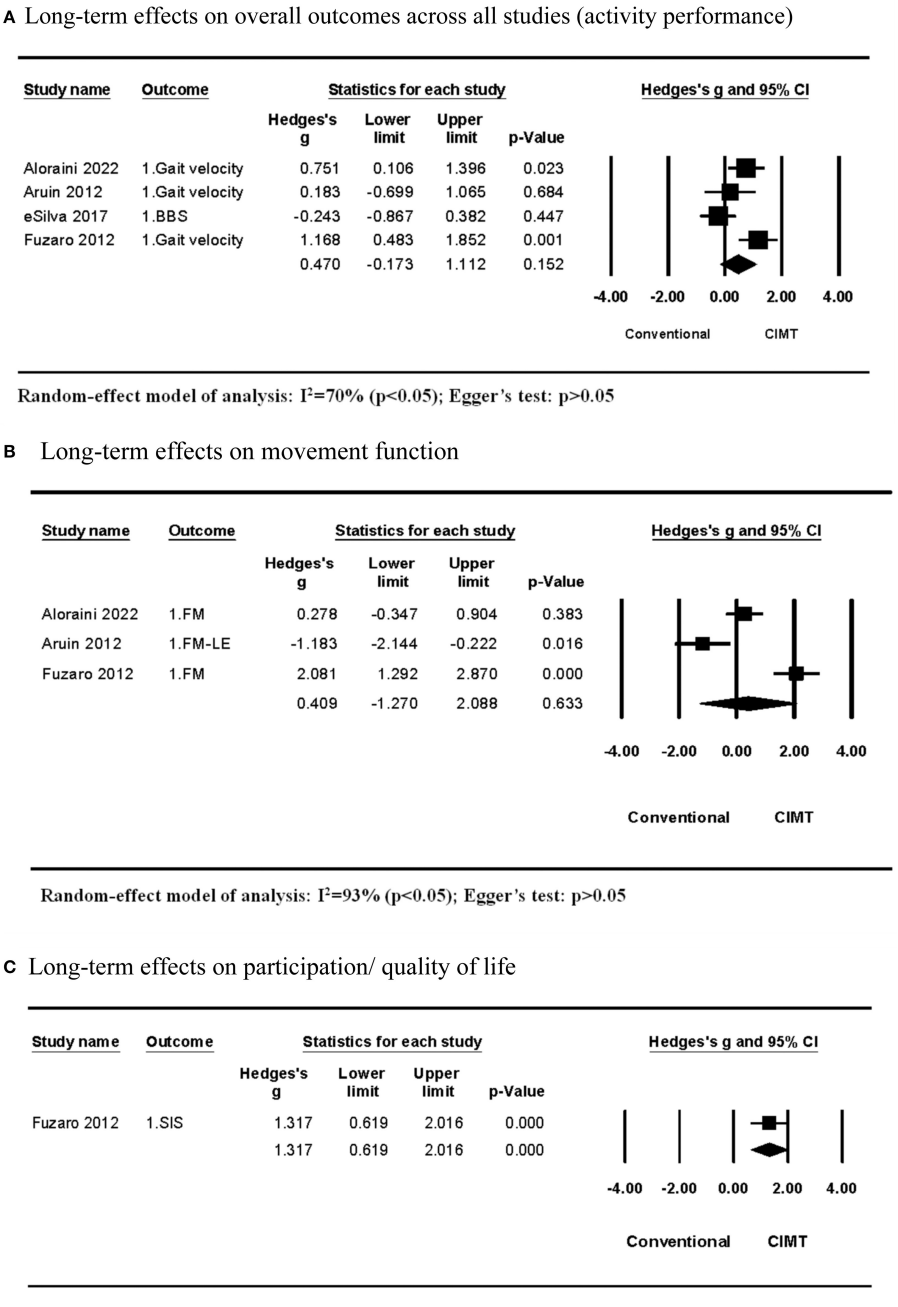
Long-term effects of CIMT compared with conventional treatment. **(A)** Long-term effects on overall outcomes across all studies (activity performance). **(B)** Long-term effects on movement function. **(C)** Long-term effects on participation/quality of life.

Four studies compared the long-term effects between CIMT and conventional treatment. All of them examined the effects on the activity outcome, while three of them explored the effects on the movement function as well. The meta-analysis of studies examining effects on the activity outcome discovered that CIMT had a small but insignificant effect size compared with conventional treatment (Hedges' g = 0.470; *P* > 0.05; 95% CI: −0.173 to 1.112) and moderate heterogeneity across studies (*I*^2^ = 70%; *P* > 0.05) (Figure 3A). In single-variable meta-regression analysis, the CIMT method using a weight strapped around the non-paretic leg (β = −1.000, *R*^2^ = 77%, *P* > 0.05) had a significant contribution (Table 4). Total training time contributed significantly to single-variable meta-regression analysis (β = 0.002, *R*^2^ = 53%, *P* > 0.10) but was unable to be included further mixed-variable analysis due to the insufficient number of studies.

The meta-analysis across studies examining effects on the movement function discovered that CIMT had a smaller but insignificant effect size than conventional treatment (Hedges' g = 0.409; *P* > 0.05; 95% CI: −1.270 to 2.088) and large heterogeneity across studies (*I*^2^ = 93%; *P* > 0.05) (Figure 3B). The CIMT methods were different across three studies. They used a wedged insole under the non-paretic foot, task-specific force-use therapy, and an arm sling to constrain non-paretic arm movement. The between-subgroup analysis revealed that the effect size of the CIMT method using a wedged insole under the non-paretic foot compared with conventional training was significantly lower than other CIMT methods, whereas the CIMT method using an arm sling was significantly higher than others (Table 4).

One study by Fuzaro et al. examined the long-term effects on participation or the quality of life. They discovered that CMT had a larger effect size than conventional treatment (Hedges' g = 1.317; *P* > 0.05; 95% CI: 0.619 to 2.016) (Figure 3C).

Egger's test revealed no publication bias across studies included in each meta-analysis on long-term effects (*P* > 0.05; Figure 3).

### 3.4. Comparisons between CIMTs

**Figure 4:**
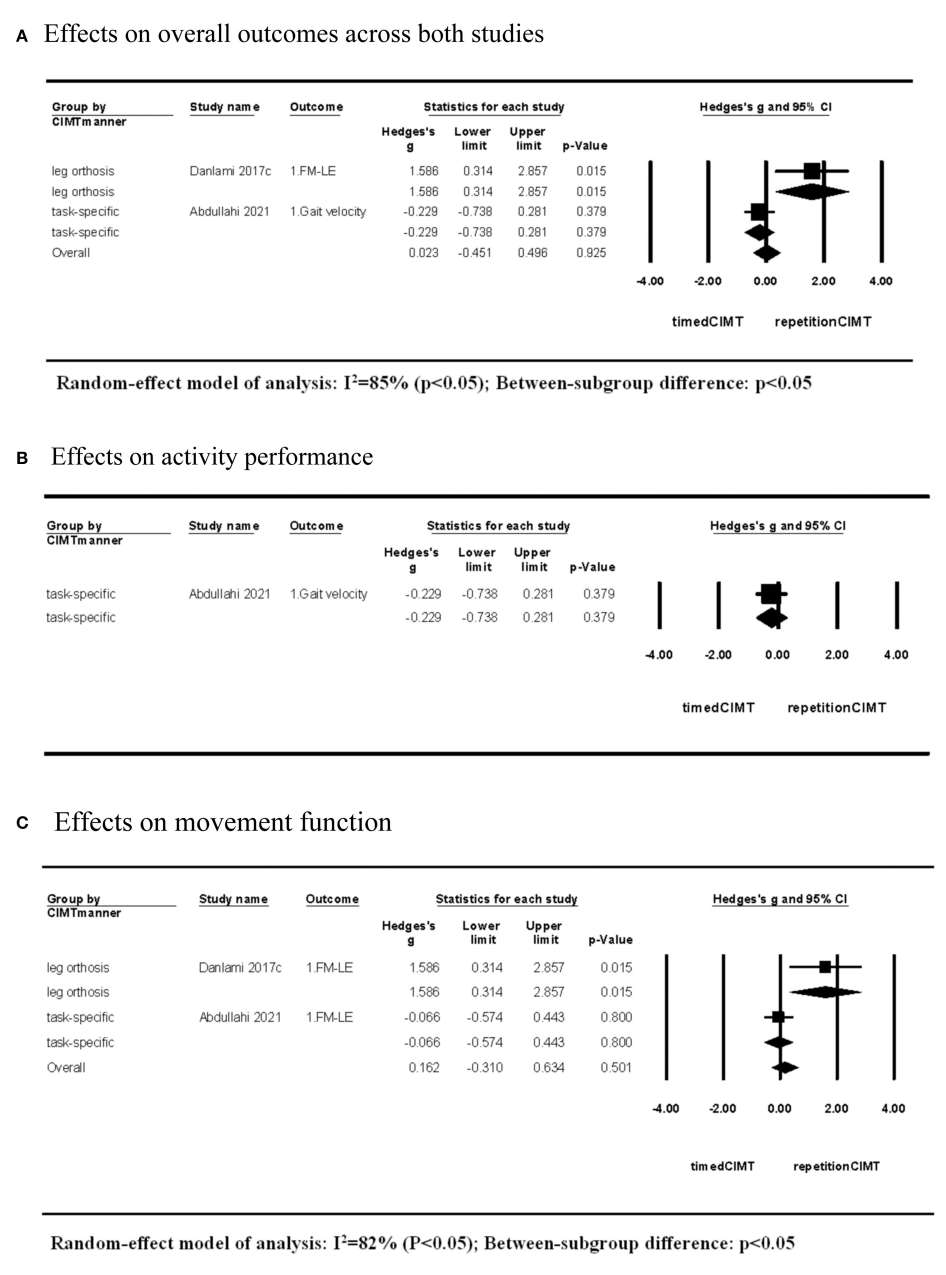
Short-term effects of CIMT with dosage quantified by repetitions compared with that quantified by time. **(A)** Effects on overall outcomes across both studies. **(B)** Effects on activity performance. **(C)** Effects on movement function.

Two studies compared the short-term effects of CIMT quantified by repetitions with that quantified by time (6, 10). The CIMT using an orthosis to constrain non-paretic leg movement showed superior effects on movement function when setting dosage with 960 repetitions compared with the dosage of 40 h. In contrast, CIMT using task-specific force-use therapy demonstrated no difference in effects on activity performance and movement function between dosages of 12,000 repetitions and 60 h. The CIMT method showed significant between-subgroup differences in the effect size (*P* > 0.05; Figure 4).

## 4. Discussion

This is the first systematic review to investigate the influence of the CIMT method on treatment effects of improving lower limb outcomes, considering participant characteristics, CIMT features, and ICF outcome categories. Based on studies comparing CIMT and conventional treatment, CIMT produced larger effects on lower extremity-relevant activity performance, movement function, and participation in the short-term than conventional treatment but with different levels of heterogeneity. Meta-regression discovered that the lower limb movement function is more sensitive to improvement than activity performance by CIMT. The CIMT method using a weight strapped around the non-paretic leg had poorer effects on outcomes of activity performance or movement function of the lower extremity than other CIMT methods. In contrast, the method using an arm sling had more prominent effects on the movement function of the lower extremity than other CIMT methods. In the long-term, CIMT had insignificant effects on the activity performance and movement function, with moderate to large heterogeneity. The CIMT method using a weight strap or an arm sling played similar contributing roles on long-term effects as the roles on short-term effects. Participant characteristics had no effect on the training effects of CIMT. Based on studies comparing different CIMTs, CIMT using an orthosis with a dosage quantified by repetition had superior effects on the movement function than that with a dosage quantified by time. These findings could help guide clinical applications of CIMT to improve lower limb outcomes in individuals after stroke.

Two previous review studies focused on CIMT effects on lower limb outcomes (30). The first review by Abdullahi et al. (30) included six studies in a meta-analysis and discovered no superior effects on all outcomes except for the quality of life produced by CIMT relative to active control groups. Tedla et al. (15) discovered that CIMT is superior to the active control group in improving the balance ability but not functional mobility, including more studies and using subgroup meta-analysis. Our study did not differentiate balance ability measured by using the BBS from functional mobility measured by the walking test, considering that BBS is an instrument used to evaluate functional mobility on sitting, standing, and positional or postural transfer, and it correlates with gait velocity (31). We categorized outcomes based on the ICF framework, a widely used method in meta-analysis studies, ensuring that more studies are included in subgroup meta-analyses (4, 24). Unlike previous reviews, our study discovered that CIMT had superior short-term effects on activity performance, movement function, and participation than the conventional treatment. Thus, the number of CIMT studies and the proper outcome category are important in determining meta-analysis results across studies.

Despite the superior effects of CIMT relative to conventional treatment on overall outcomes or outcomes of each ICF category from meta-analysis, moderate to high heterogeneity existed across studies of each meta-analysis. Participant characteristics, CIMT methods, and outcome categories could be potential factors contributing to heterogeneity. CIMT using an arm sling has been found to have greater effects on the upper limb motor function at early stages after stroke; however, improved performance of the upper limb-related activity is more at subsequent stages (4). This could be attributed to cortical reorganization, which prominently occurs at an early stage after stroke, correlating with motor function improvement (23). However, our study demonstrated that CIMT had greater effects on the motor function of the lower limb than on activity performance no matter at any stroke stage, by the meta-analyses across all studies and across studies of each outcome category. Participant characteristics had no contribution to effect sizes across studies involved in each meta-analysis. One explanation is that CIMT involves more movement or weight bearing of the paretic lower limb in training tasks or activities but does not specifically increase the activity amount of the paretic lower limb like increasing upper limb activity amount using upper limb CIMT. Therefore, the lower limb movement function improves more than activity performance.

Moreover, CIMT methods are the main factors contributing to the heterogeneity of effect size across studies, which we aimed to clarify in this study. We discovered that the CIMT method using a weight strapped around the non-paretic leg had a poorer effect on outcomes of the activity performance or lower extremity movement function than other CIMT methods. The weight strapped around the non-paretic leg could not constrain the non-paretic leg, but it could increase the sensory input and efforts of the non-paretic leg against the resistance during training tasks, conversely reducing the involvement of the paretic leg. Other methods, including using a wedged insole, leg orthosis, arm sling, or task-specific force-application therapy, limit the movement or weight bearing of the non-paretic leg, resulting in better outcomes after stroke. Moreover, the CMIT method using an arm sling had more prominent effects on the movement function than other methods. This result must be cautiously interpreted because the movement function was measured using the full FMA scale in two of three CIMT studies. Therefore, the prominent effects on movement function might be unrelated to the lower limb function.

The CIMT method and dosage are important factors in ensuring treatment effectiveness. This review only included studies with dosage-matched conventional training as the active control group and excluded those with a pure control study. The selection criterion minimized the heterogeneity of effect size resulting from dosage. Thus, the dosage had no influence on effect sizes across studies comparing CIMT and conventional treatment in this review. Interestingly, we found two studies exploring the influence of the dosage quantified method of CIMT on outcomes when we included studies with different CIMTs. Dosages by time are the most common quantification method of CIMT. In contrast, the repetition of practice is a more rigorous quantifying method (32) because the number of task repetitions required for motor recovery is more clear, including at least 300 repetitions per day (20). We discovered differences in effects between the two dosage methods in the CIMT using an orthosis to constrain non-paretic leg movement but not in CIMT using task-specific force-use therapy. Physical constraints using an orthosis could reduce participants' initiative and decrease completion efficacy, which could not occur in CIMT without physical constraints. Therefore, the number of task repetitions guarantees the completion volume better than the time for CIMT with physical constraints.

Three-step meta-regression is a strict method for determining the best regression model to investigate factors contributing to the variation in effect size across studies, with reference to the procedure of multiple linear regression (26). Using the between-subgroup analysis, results of the between-subgroup difference could not only directly attribute to the factor based on which subgroups are divided but also to other factors which are different between subgroups by chance. Therefore, the interaction between multiple factors should be considered in exploring the influence on treatment effects. In our study, age showed a nearly significant negative correlation with the effect size of CIMT in single-variable regression analysis which is similar to the between-subgroup analysis. However, it did not contribute significantly to the mixed-variable regression model with other factors. Thus, age was excluded from the final regression model of explaining the variation in effect size across studies. The example suggests that age influences the effect size occasionally. The strict meta-regression ensures the reliability of our results.

The long-term effects of CIMT were insignificant on activity performance and movement function, with moderate to high heterogeneity across studies. The CIMT method using a weight strapped around the non-paretic leg and an arm sling similarly contributed to the long-term effect size. However, the result should be cautiously interpreted because of the small number of studies, leading to an inapplicability of the restricted mixed-variable regression analysis. Long-term effects correlate with the transfer package of the upper limb during CIMT (33). Among studies exploring the long-term effects of lower limb CIMT, CIMT using a weight strapped around the non-paretic leg did not adopt a transfer package (9); however, other studies encouraged the use of force tasks in daily life or continued using CIMT constraints setting in daily life (7, 11, 14). Therefore, the transfer package could also be a contributing factor. However, the amount of transfer package or the behavior change from lower limb CIMT during follow-up has not been reported, which should be included in future studies to clarify the mechanism underlying long-term effects.

Despite its meaningful findings, this study had several limitations. First, methodological quality evaluated by Cochrane risk-of-bias revealed potential sources of bias in all included studies. Second, studies were inadequate for exploring both short-term and long-term effects on the participation outcome after stroke and for performing mixed-variable meta-regression in exploring contributing factors of the effect size of CIMT relative to conventional training. Third, based on the characteristics of participants, the findings of this study cannot be generalized to all patients with stroke, such as those with cognition impairments or in the acute stage after stroke. Studies with high methodological quality, large sample sizes, and targeting different stages after stroke are required to further explore the effects of lower limb CIMT and the contributing factors of its effects on outcomes.

## 5. Conclusion

Constraint-induced movement therapy is superior to conventional treatment in improving lower limb activity performance, movement function, and participation in the short-term but not in the long-term. The CIMT method using a weight strapped around the non-paretic leg had poorer effects on the activity performance and movement function of the lower limb, than other CIMT methods. The method using an arm sling had a more prominent effect on the movement function than other CIMT method, which should be interpreted with caution. The repetition of task practice is a superior dosage quantification method to practice time in CIMT with physical constraints. These findings could help guide clinical applications of CIMT to improve lower limb outcomes in individuals after stroke.

## Data availability statement

The original contributions presented in the study are included in the article/supplementary material, further inquiries can be directed to the corresponding author.

## Author contributions

JZ and XS: study objective. JZ and HF: literature search and data extraction. JZ, HF, and JL: methodological quality assessment. JZ, HF, JL, HZ, and XS: critical review and approval of manuscript. All authors read and approved the final manuscript.
